# Non-Coding RNAs: Strategy for Viruses’ Offensive

**DOI:** 10.3390/ncrna6030038

**Published:** 2020-09-10

**Authors:** Alessia Gallo, Matteo Bulati, Vitale Miceli, Nicola Amodio, Pier Giulio Conaldi

**Affiliations:** 1 Department of Research, IRCCS ISMETT (Istituto Mediterraneo per i Trapianti e Terapie ad alta specializzazione), Via E.Tricomi 5, 90127 Palermo, Italy; mbulati@ismett.edu (M.B.); vmiceli@ismett.edu (V.M.); pgconaldi@ismett.edu (P.G.C.); 2 Department of Experimental and Clinical Medicine, Magna Graecia University of Catanzaro, 88100 Catanzaro, Italy; amodio@unicz.it; 3 UPMC Italy (University of Pittsburgh Medical Center Italy), Discesa dei Giudici 4, 90133 Palermo, Italy

**Keywords:** immune evasion, miRNA mimicry, viral circRNA, viral non-coding RNA

## Abstract

The awareness of viruses as a constant threat for human public health is a matter of fact and in this resides the need of understanding the mechanisms they use to trick the host. Viral non-coding RNAs are gaining much value and interest for the potential impact played in host gene regulation, acting as fine tuners of host cellular defense mechanisms. The implicit importance of v-ncRNAs resides first in the limited genomes size of viruses carrying only strictly necessary genomic sequences. The other crucial and appealing characteristic of v-ncRNAs is the non-immunogenicity, making them the perfect expedient to be used in the never-ending virus-host war. In this review, we wish to examine how DNA and RNA viruses have evolved a common strategy and which the crucial host pathways are targeted through v-ncRNAs in order to grant and facilitate their life cycle.

## 1. Introduction

Since 1964, when Epstein discovered, in pediatric cases of Burkitt’s lymphoma, the first human virus linked to malignancy, the scientific attention focused on the viral regulatory mechanisms within host cell capable to create the perfect cellular environment for viral replication. Since then, it has been postulated that the 12% of all human cancers have a viral etiology [1]. Also, novel emerging viruses, as SARS-CoV-2 and Zika, that spill over from wildlife into domestic animals and humans, are becoming a constant threat for human public health [2]. The fact that viruses dramatically limit their genomes size to the minimum number of genes for infection [3], suggests that viruses and their hosts have been involved in a never-ending struggle of adaptation defined by a plethora of complex mechanisms [4]. The crucial and central point is that the viruses are perfect parasites [5] and cannot survive without a host cell providing the transcriptional machinery. On the other side, the eukaryotic cell, does not freely allow parasites to enter, so the viruses have developed many different mechanisms in order to skip the host cell barrage, beginning with the recognition of surface molecules acting as key door, and continuing with fundamental immune evasion, all aimed at translocating proteins and genetic material into the cells. On that note, it raises that viruses are not alive stricto sensu but, even if they are made of the simplest structure existent, they result the cleverest infectious agents because of the ability to adjust themselves, use and trick the host.

The first step for a virus, either enveloped or non-enveloped, after initial attachment to the cell surface, is the penetration in the host by both membrane fusion and endocytosis [6]. Once inside the host, a virus particle uncoats its capsid and releases the naked viral genome in order to establish gene expression and viral genome replication [7]. It has been postulated that since the viral genome is size-limited, any non-coding space is rationated [8]. Recently, thanks to the novel high-throughput RNA sequencing techniques it has been discovered that many, but not all viruses genome express non-coding RNAs (ncRNAs) for their own benefit [9]. According to the Baltimore classification (first defined in 1971), viruses are divided into seven categories depending on the nucleic acid (DNA or RNA), strandedness (single-stranded or double-stranded), sense, and method of replication [10]. The viruses that have include the majority of ncRNAs belong to a DNA virus family (herpesviruses), perhaps because they have relatively large DNA genomes [8] capable to produce RNA intermediates [11].

RNA viruses show a great intrinsic epidemic potential [12] due to the high mutation rates and high frequency of recombination events. Surprisingly, it has been discovered that also RNA viruses produce ncRNAs whom expression is highly correlated with viral infection activity [13].

The reason why the ncRNAs, and in particular viral ncRNAs, are earning more interest rely on their functions as regulators of translation, RNA splicing, and gene expression [14] and because of the important impact they can exert on host pathways. NcRNAs belong to a very heterogeneous family, in terms of length, conformation and cellular function [15]. At present, ncRNAs can be divided by length (cut-off 200 bp) into two main groups: long non-coding RNA (lncRNA) and small non-coding RNA (sncRNA). LncRNA can be further grouped into linear RNAs and circular RNAs.

Linear lncRNA molecules are at least 200 nucleotides long, often harbor a poly-A tail and can be spliced, as mRNAs, but lack of protein-coding potential [16]. LncRNAs are generally nuclear localized, demonstrating their role as regulators of nuclear organization and function [17]. Circular RNAs (circRNAs) are a novel class of non-coding RNAs, whose main characteristic is the covalently closed loop structure without terminal 5ʹ caps and 3ʹ polyadenylated tails [18]. Since circRNAs contain miRNA response elements (MREs), it has been suggested they act as miRNA sponges through competitive binding to miRNAs, with a consequent weakening of mRNAs regulation [19].

Small non-coding RNAs are a class of non-coding RNA composed mainly by microRNAs (miRNAs), piwi-interacting RNAs (piRNAs), small nucleolar RNAs (snoRNAs), and recently discovered tRNA-derived RNA fragments (tRFs) [14]. MiRNAs negatively affect mRNA protein output by imperfectly base pairing with the 3′-untranslated region (3′UTR) [20]. The complexity of this mechanism lies in the combinatorial mode of miRNA action in mRNA regulation, since a single miRNA is able to target several mRNAs [21].

All the ncRNAs classes described above have been extensively studied in mammals, but since the first v-ncRNA was discovered, the v-ncRNAs become extremely appealing because of the role played in all the infection steps. V-ncRNAs are gaining as much pathological importance as viral structural proteins [22]. In this review, we intent to examine how viruses have evolved a common strategy and which are the crucial host pathways targeted through v-ncRNAs in order to grant and facilitate their life cycle.

## 2. Viral Immune Evasion Strategies

The first step of the infection process is the evasion from the host immune surveillance system. This difficult task to achieve, is a complex balance between limiting viral gene expression in order to limit antigen presenting molecules [23] and producing viral immune evasion against innate and adaptive immunity [24].

Since ncRNAs are not presented via Major histocompatibility complex (MHC), they result to be non-immunogenic to the adaptive immune system and thus particularly useful tools for viruses to influence host cell functions [25]. Interestingly, viruses produce v-ncRNAs capable of controlling not only the expression of viral genes, but also influence host cell regulation and evade host innate and specific immune responses, crucial mechanisms in the viral pathogenetic processes [9]. Humans are equipped of pattern recognition receptors (PRRs), such as Toll-like receptors (TLRs), retinoic acid-inducible gene-I (RIG-1), and protein kinase R (PKR), that are able to detect foreign nucleic acids, as viral RNAs [24]. Nevertheless, viruses have developed ncRNA traps to escape detection. As an example, viral-derived dsRNA induce the activation of both dsRNA dependent-PKR and TLRs, which results in type I IFN response, a well-known anti-viral defense mechanism [26]. Different v-ncRNAs, like EBV’s Epstein-Barr virus-encoded small RNAs (EBERs), adenoviral VAI and VAII, and HIV’s trans-activation response RNA (TAR) act as a trap to inhibit PKR activation [9]. The inhibition of IFN production is one of the most important strategies developed by viruses to block antiviral response. Indeed, it has been recently found that IFN signaling is one of the main pathway regulated by virus-derived lncRNAs. Polyadenylated nuclear RNA (PAN) from Kaposi Sarcoma Herpes virus/Human Herpesvirus-8 (KSHV/HHV8), during its lytic phase of infection, interacts with IRF4 inducing reduced expression of IFNα and IFNγ [27]. A common feature of flavivirus is the production of viral non-coding subgenomic RNAs derived from partial degradation of the viral genome, known as subgenomic flavivirus RNAs (sfRNAs), which are involved in immune evasion. Among flaviviruses, Zika virus produces two different sfRNA, sfRNA1 and sfRNA2, which inhibit IFN production by targeting STAT2 pathway [28,29].

Moreover, sfRNAs produced by numerous flaviviruses, including Japanese encephalitis virus (JEV), West Nile Virus (WNV) and dengue virus antagonize the antiviral response by inhibiting the IFN signaling, the expression of IFN-β or specific IFN-stimulating genes (ISGs) [30]. Additionally, a chimeric lncRNA HBx-LINE1, which derives from the integration of HBV into host cell genome, attenuate the IFN antiviral response inhibiting the host cell-derived miR-122 [30]. Together with the ability to inhibit type I IFN antiviral response, v-ncRNAs are able to influence cytokines and chemokines secretion as a mechanism of immune evasion. On this purpose, ebv-miR-BART6-3p acts in two different ways: it can directly binds to RIG-1 mRNA, with consequent impaired production of the antiviral cytokine type I IFN [31], or in association with host-derived miR-197, acts on IL-6R mRNA induced an impaired production of the pro-inflammatory cytokine IL-6 [32]. Another EBV-derived miRNA that inhibits type I IFN signaling is ebv-miR-BART16, which has as specific target the host mRNA CREBBP [33]. Similarly, KSHV/HHV8 produces kshv-miR-K12-11, which induces the impairment of type I IFN signaling by targeting IKKε [34], and kshv-miR-K12-10 which reduces the production of IL-6 and IL-10 by targeting TWEAKR [35]. Ebv-miR-BHRF-1-2-5p and ebv-miR-BART15 acts on IL-1 signaling, the former by targeting IL-1 Receptor 1 [36] and the latter inhibiting IL-1β production [37]. Two components of TLR/IL-1R-mediated signaling, MYD88 and IRAK1, are, respectively, the targets of the KSHV/HHV8 derived miRNAs kshv-miR-K12-5 and kshv-miR-K12-9, which affect the secretion of inflammatory cytokines [35].

In addition, also the MHC-restricted antigen processing and presentation can be influenced by v-ncRNAs. Indeed, ebv-miR-BART2 interferes with MHC-I antigen processing because of it targets CTSB mRNA, while ebv-miR-BHRF1-3 blocks peptide transport to MHC-I, targeting TAP2 [38], and ebv-miR-BART1-5p inhibits antigen capture and processing acting on LY75 mRNA [39]. Both ebv-miR-BART1 and ebv-miR-BART2, linking respectively to IFI30 mRNA and LGMN mRNAs, induce the impairment of MHC-II-restricted antigen processing [39]. Finally, an in silico analysis demonstrated that a Merkel Cell Polyomavirus (MCPyV)-derived miRNA, namely MCV-miR-M1-5p, seems to direct targeting an intrinsic antiviral protein, SP100, which leads to a reduction in the secretion of CXCL8 with a final effect of the subversion of the host-cell immune response, influencing neutrophils chemotaxis [40].

NcRNAs can also influence host T cell behavior. In fact, two HPV-derived miRNAs, targeting different host mRNAs, such as BCL11A, CHD7, ITGAM, RAG1, and TCEA1 (miR-H1-1) or PKNOX1, SP3, XRCC4, JAK2, and FOXP1 (miR-H2), are able to inhibit T cell development and activation [41]. Additionally, many EBV-derived miRNA target genes involved in T cell polarization and migration, namely ebv-miR-BART1, -BART2, -BART10, -BART22, and -BHRF1, prevent the polarization of CD4^+^ T helper cells toward antiviral Th1 subtype acting on IL12B mRNA [36,38], whereas BHRF1-3 inhibits CXCL11 mRNA, blocking the chemotaxis of activated T cells [42]. More recently, it has been suggested a role of polyomaviruses’ ncRNAs in the modified behavior of T cells during infection. Indeed, the beta-polyomaviruses John Cunningham virus (JCV), BK virus (BKV), simian virus 40 (SV40) and MCPyV encode two miRNAs (miR-S1 and miRJ1), which control the viral replication by inhibiting viral T antigen expression that lead to the suppression of antiviral T cell response [43]. Interestingly, different HIV-1-derived ncRNAs are able to suppress Anti Sense Protein (ASP), which normally induces CD8 T cell responses during chronic infection [44], so inhibiting CD8 T cells activation and functioning [45]. Although the detection of v-ncRNAs in RNA viruses is controversial [46], other than HIV retrovirus, also a negative-sense RNA virus as Ebola, is able to produce miRNA-like small RNAs [47]. Liu et al. described as EBOV-MiR-1-5p, an analog of host miR155, inhibits the expression of importin-α5, which seems to be a potent mechanisms of immune evasion [48].

Some v-ncRNAs are able to induce NK cell evasion of virally infected cells by inhibiting the action of NKG2D. This receptor exert its killing function by recognizing stress-induced ligands, such as MHC class I-related chain B (MICB) or UL16 binding protein 3 (ULBP3), which are upregulated on virally infected cells. In particular, it has been observed that miRNAs encoded by different herpes viruses, such as hcmv-miR-UL112-1 (CMV), ebv-miR-BART2-5p (EBV) and kshv-miR-K12-7 (KSHV), inhibit the NKG2D action by targeting MICB [49,50,51]. Besides, a miRNA conserved between two different polyomaviruses (JCV and BKV), namely miR-J1-3p, targets the stress induced molecule ULBP3 [52].

**Figure 1:**
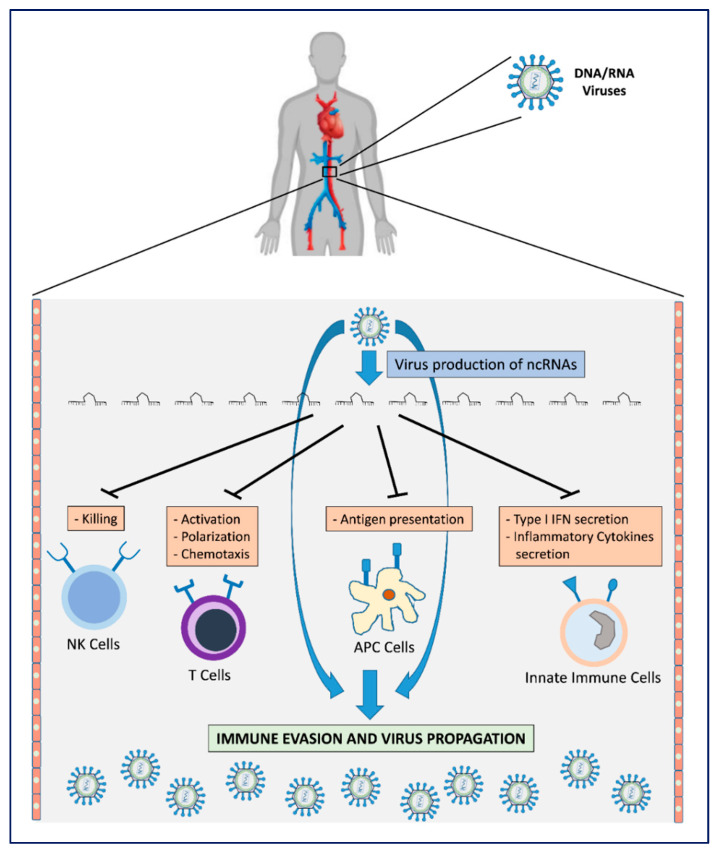
Viral mechanisms for host immune surveillance evasion by production of v-ncRNAs. The production of v-ncRNAs acts on the main mechanisms by which host immune effector cells oppose virus infection. This leads to escape from immune recognition with consequent virus proliferation/propagation.

As described, different viruses, despite encoding completely different ncRNA sequences, always share common targets and mechanisms of immune evasion (Table 1), opening an important question about the co-evolutionary development of v-ncRNAs and their matching cellular targets (Figure 1).

## 3. Viral Non-Coding RNAs as Transcriptional Weapons

**Figure 2:**
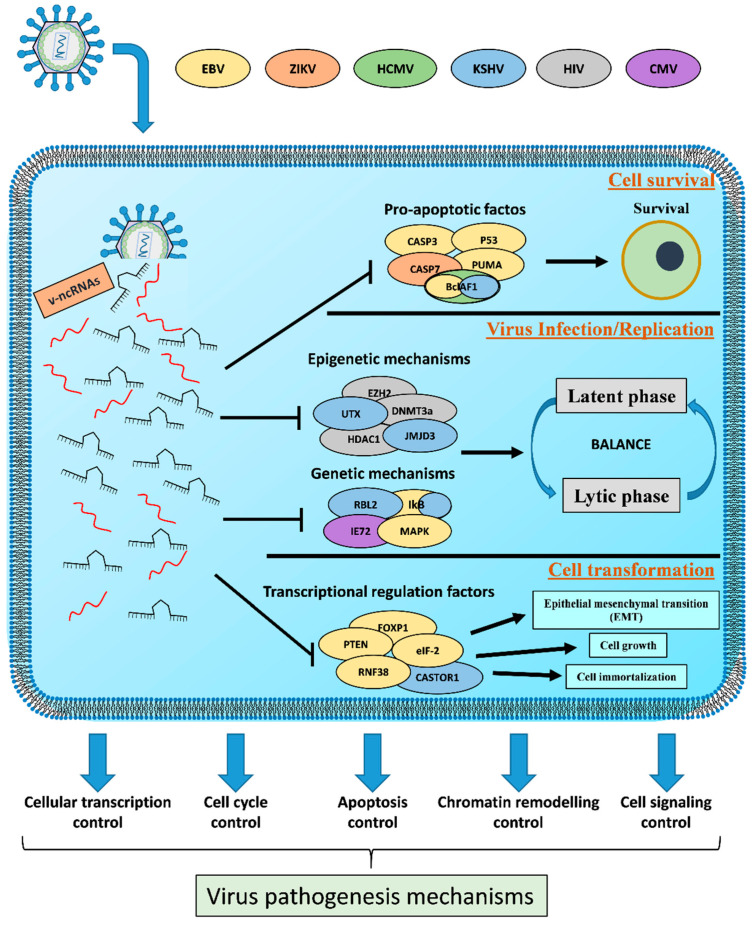
Role of v-ncRNAs in the virus pathogenesis mechanisms. V-ncRNAs act as fine regulators between the latent and lytic phase and as regulators of both survival and cell transformation. The control of these host physiological processes contribute to virus replication/propagation and cell pathogenesis.

Several viruses are able to produce v-ncRNAs that are frequently expressed at high copy numbers in infected cells [8]. V-ncRNAs are capable of interacting with different host cell pathways leading to the modulation of different biological processes including: 1. regulation of viral and host gene expression [53,54]; 2. cell survival [53,54]; 3. viral infection/replication [55,56]; 4. cell transformation [54,57]; 5 virus proliferation/propagation [58,59] (Figure 2). On the other hand, host cells regulate their own ncRNAs expression in order to activate defense mechanisms against virus infection.

### 3.1. Regulation of Viral and Host Gene Expression

As already stated, viruses usurp the host transcriptional machinery to ensure their survival and the majority of viral mRNAs is synthetized by host RNAPII [4,60]. Moreover, it has been demonstrated that some v-ncRNAs, synthetized by DNAviruses, such as adenovirus-derived virus-associated RNAs (VA RNAs) and Epstein-Barr early RNAs (EBERs), are transcribed by host RNAPIII, with the characteristic of being not widespread rather expressed at high copy numbers in infected cells [8]. A different scenario involves the RNAviruses, double stranded (ds) and single positive/negative stranded (+ss or -ss, respectively), which encode for a RNA-dependent RNA polymerase (RdRp) [61]. Furthermore, some v-ncRNAs are not generated from canonical pathways; they instead derive from degradation of a unique viral mature sequence processed by host cellular machineries. For example, flavivirus RNA is degraded by RNases exoribonuclease 1 (XRN1) in host cells and this process represents a defense mechanism. In this regard, flavivirus has developed a particular RNA structure to alter XRN1 activity and to produce a large amount of degraded intermediates termed sfRNAs. These molecules are considered v-ncRNAs operating in the virus infection [13]. In the last decade, it has emerged the concept that the sequences discarded during splicing (stable intronic sequence RNAs, sisRNAs) may play physiologic roles, and this phenomenon appears to be very suitable for viruses that naturally have genomes with limited dimensions. Indeed, v-ncRNAs comprise of sisRNAs, were first discovered in the herpes simplex virus 1 (HSV-1) [62]. During latency phase, HSV-1 produces high quantities of LAT ncRNA and its respective excised introns persist and accumulate to high levels in infected cells acting as sisRNAs [62,63]. Although negative-sense RNA viruses do not replicates in the nuclear compartment, it has been shown that also human metapneumovirus (hMPV) produce several v-ncRNAs and, due to the fact that the transcription and replication of hMPV occur in the cytoplasm, cytoplasmic RNase as the XRN1 may be involved in the biogenesis of hMPV-derived ncRNAs [58,59]. In addition, different negative-strand RNA viruses including vesicular stomatitis virus (VSV), rabies virus (RABV) and influenza A virus (IAV), produce subgenomic v-ncRNAs. They were shown to interact with the viral RNA polymerase to regulate the switch from mRNA synthesis to viral genome replication influencing viral life cycle [64,65].

V-ncRNAs act as substrates for RNase Dicer, with the products being incorporated into argonaute-containing RNA-induced silencing complexes (RISCs) [66]. As a consequence of this process, and due to the high copy number of v-ncRNAs in infected cells, cellular miRNA biogenesis may be significantly altered [67]. Another herpesvirus that produce v-ncRNAs is Herpesvirus Saimiri (HVS) [68,69]. Recently, different v-ncRNAs derived from HVS were discovered to interact and down-regulated host miRNAs including miR-27, miR-16, and miR-142-3p and, with an antisense RNA-based mechanism [70].

It has been also demonstrated that some viruses use v-ncRNAs as scaffold for transcriptional factors recruitment. The EBV encoded EBER2, for example, acts as a transacting guide to promote its own transcription [71]. Collectively, all the above-reported mechanisms are just some examples of how viruses have developed strategies with the final scope of taking over the transcriptional machinery and promote viral replication.

### 3.2. Host Cell Survival

As viruses are perfect parasites, it seems obvious that killing the host is not the best strategy for self-propagation. For this reason, viruses have developed different ways in order to influence host cell survival and block apoptosis as essential components of the cell response to injury [72]. Among Adenovirus Virus-Associated (VA) RNAs, mivaRNAI-138 can inhibit TIA-1 mRNA, a well known factor that activates apoptosis [73]. Moreover, VA RNA I is involved in the selective translation of viral mRNAs and suppression of host cell protein translation. Indeed, this ncRNA inhibits both the cleavage of double-stranded RNA and the protein kinase R (PKR), which when activated, is responsible for the phosphorylation and activation of eIF-2 (a factor capable of inhibiting protein synthesis in cells infected by virus) [74]. The latency associated non coding transcript (LAT) coded by herpes simplex virus 1 (HSV-1), exerts anti-apoptotic effect [75] and inhibits the expression of viral early genes to maintain latency by down regulating both TGF-β1 and SMAD3 expression [76]. LAT seems to play a crucial role also for Herpes simplex virus 2 (HSV-2). Indeed, it has been demonstrated as HSV-2-produced LAT inhibits apoptosis and maintains latency via LAT-encoded microRNAs (miR-H3, miR-H4-3p, miR-H4-5p, miR-H24 and miR-H19) providing protection against apoptosis induced by ActD [77]. EBV miRNAs can give a growth advantage in infected cells and therefore contribute to cell transformation both in vitro and in vivo [78,79,80]. In particular, pro-apoptotic genes like CASP3, PUMA and P53 are EBV’s miRNA targets [81,82,83]. CASP3 is also a reported target of KSHV miRNAs, miR-K12-1, K12-3 and K12-4-3p, thus reducing apoptosis [84]. Kshv-miR-K12-1 targets p21, a key tumor suppressor and inducer of cell cycle arrest, controlling cell survival and proliferation [85]. Another DNA virus, MCPyV produces a 2.7-kb RNA (β2.7) capable of preventing mitochondria-induced apoptosis [86]. Besides, it has been demonstrated that in infected mosquitoes by ZIKV, member of Flaviviridae family, the presence of sfRNA facilitates ZIKV infection and transmission by inhibiting apoptosis through the regulation of CASP7 [87]. It is also remarkable that human CMV, EBV, and KSHV have been shown to encode miRNAs, hcmv-miR-UL112-1, ebv-miR-BART-17-5p and kshv-miR-K5 respectively, which target the same pro-apoptotic gene: BclAF1 [88,89,90]. The miRNAs target sites are different for each v-miRNAs underlying the crucial role that BclAF1 plays in the life cycle of diverse herpesviruses and how different viral miRNAs may converge on similar targets without depending on the same conserved target sites [91].

### 3.3. Viral Efficient and Persistent Infection Regulation

Once the virus has took over the translational machinery and has assured the life host mainteinance, the following step is to maintain itself in a replicating yet not distruptive infectious status. HIV-1 v-ncRNAs, by repressing the polycomb gene EZH2, the DNA methyltransferase DNMT3a and the histone deacetylase HDAC1, modulate HIV-1 latency through epigenetic modulation [45,92]. The expression of HSV-1 sisRNAs, maintains viral infection by inhibiting apoptosis and silencing viral lytic gene expression through modification of the viral promoters [93,94]. In human and murine citomegalovirus (CMV) other sisRNAs have been identified, and these molecules seem to be involved in the progression from acute to persistent infection [95]. Recently, sisRNAs were identified also in Epstein–Barr virus (EBV) [96] and these molecules, including EBV sisRNA-1, were related with oncogenic latency processes [97]. In human B cells infected with KSHV, v-ncRNAs are mainly represented by nuclear polyadenylated (PAN) RNAs [98,99] that interact with demethylases JMJD3 and UTX [100]. Furthermore, it has been shown that PAN RNA also binds to the KSHV latency-associated nuclear antigen (LANA), and this interaction could be involved in the virus reactivation from latency phase [101]. Besides, hcmv-miR-UL70 may regulate MAPK signaling, focal adhesions and gap junctions’ pathways affecting epithelial cell migration and adhesion [102]. Hcmv-miR-UL112 downregulate the major immediate–early gene IE72 leading to latency through a decreased expression of viral genes involved in replication [103]. Moreover, hcmv-miR-UL112 induces proliferation of endothelial cells by up-regulating MAPK pathways or genes involved in cell growth including TSPYL2, FXYD2, TAOK2, ST7L, and TP73 [104,105]. The latter mechanism could represent the way by which human CMV induces endothelial dysfunction in CMV-mediated vascular diseases. Furthermore, ebv-miR-BART8-3p and ebv-miR-BART13 targets RNF38 (an E3 ubiquitin-protein ligase able to ubiquitinate p53) and NKIRAS2 (NF-kB Inhibitor) respectively [106,107,108], whereas ebv-miR-18-5p suppresses MAPK signaling by targeting MAP3K2, with consequent regulation of lytic viral replication [109]. Also, the NF-κB pathway is regulated by KSHV’s miRNAs. Indeed, kshv-miR-K1 and kshv-miR-K12-1 inhibit viral lytic replication by down-regulating IκB and activating NF-κB signaling [110]. Kshv-miR-K12-4-5p instead targets retinoblastoma Rbl2 protein and regulates the epigenetic state of infected cells [111].

### 3.4. Cell Transformation

One of the main processes that viral infection can, either directly or indirectly, cause in host cells, is cell transformation. This process is referred to malignant transformation with typical phenotypic changes including loss of contact inhibition, acquisition of anchorage-independent cell growth, and cell immortalization. All together, these processes are favorable for the viral propagation. Therefore, viruses lead to cellular transformation by their ability to deregulate gene/protein expression with consequent alteration of cell cycle [53]. Interestingly, it has been shown that KSHV PAN RNA could maintain cellular transformation by affecting cellular gene expression that results in an enhanced growth phenotype with an increased survival [54]. In EBV, EBERs 1 and 2 regulate a variety of host cell genes including protein kinase, cell adhesion, regulation of apoptosis, and receptor signaling [112] and, in particular EBER-1 enhance host cell protein synthesis by blocking the activation of PKR [113]. Furthermore, EBER-2 but not EBER-1 plays a critical role in viral-induced growth transformation in EBV-infected B cells [57]. These findings bring to light that EBERs are potentially involved in the cell transformation in EBV-associated malignancies. Recently it has been shown that the overexpression of an EBV miRNA, ebv-miR-BART11, is involved in epithelial-mesenchymal transition (EMT) through the downregulation of FOXP1 [114], while ebv-miR-BART7-3p enhanced loss of epithelial markers and gain of mesenchymal features in neural progenitor cells (NPC) by targeting PTEN and thus affecting PI3K/Akt/GSK-3β signaling pathway [115]. Another study on NPC cells showed ebv-miR-BART8-3p plays a key role in EMT through the targeting RNF38 via the activation of NF-κB and Erk1/2 signaling pathways [107]. Furthermore, mTOR signaling, a key pathway for KSHV to induce transformation [116], is activated when KSHV miRNAs target mTOR inhibitory factor CASTOR1 [117]. Even if extremely appealing, the v-ncRNAs roles played in these processes have not being fully cleared. Much is known about viral proteins and their functions, while for v-ncRNAs, there are many descriptive yet not functional studies. So there is a plethora of studies that correlate with different viral infection the change of host ncRNAs expression rather than the v-ncRNAs. This happens mostly when the attention is focused on the EMT of the host cell, in order to identify perhaps the key passage involved in the cell transformation. For example, hsa-miR-20b, -miR-34a, -miR-218, -miR-29a and -miR-146a have been described as regulated by HPV18 E6/E7 and have been involved in initiation and progression in HPV related cervical cancer [118,119].

## 4. v-ncRNA Host Mimicry

Interestingly, viruses have evolved a clever strategy to affect directly the host pathways [91] called “mimicry”: viral miRNAs analog of host human miRNAs, with whom they share the seed sequence and potentially regulate hundreds of targets. For example, the sequence of kshv-miR-K12-11 is identical to hsa-miR-155, a host-encoded multifunctional miRNA associated with several cancers [120]. It has been shown that kshv-miR-K12-11, like human hsa-miR-155, is capable to induce B cell expansion in mice that shows an invasive phenotype in the mice spleen [121,122]. Another KHSV miRNA: kshv-miR-K6-5p shares sequence similarity to the tumor-suppressive cellular hsa-miR-15/16 miRNA family [123], resulting in an apparent nonsense mimicry, but the theory suggests the physiological role of kshv-miR-K6-5p to balance the pro-proliferative and pro-survival functions of KSHV oncogenes, negatively regulating the cell cycle [123]. Viral miRNA expression has been also shown for two retroviruses, the simian foamy virus (SFV), and the bovine leukemia virus (BLV). In particular, sfv-miR-S4-3p mimics the sequence of cellular hsa-miR-155, while sfv-miR-S6-3p mimics miR-132. Cellular hsa-miR-155 regulate cell proliferation, on this basis one could speculate that, in this case, sfv-miR-S4-3p stimulate proliferative activity of SFV infected cells [124]. It has been reported that another phylogenetically distant virus, the Marek′s disease virus, encodes for a miRNA, mdv1-miR-M4-5p, analog of hsa-miR-155, able to activate the oncogene c-Myc and to suppress the TGF-β signaling pathway [125]. Also, sfv-miR-S6-3p was shown to be a functional mimic of the IFN-suppressive hsa-miR-132, thereby helping the virus escape innate immunity. Blv- miR-B4-3p mimics the sequence of hsa-miR-29, a miRNA over-expressed in lymphoproliferative disorders, suggesting that viral miRNA expression may sustain proliferation of the infected cells playing a role in BLV associated tumorigenesis [126]. It has lately been demonstrated that a miRNA encoded by the DNA virus SV40, sv40-miR-S1-5p, has a seed sequence identical to the human hsa-miR423-5p and is able to downregulate the viral T antigen [127]. The same study has evidenced that HIV-1-encoded hiv1-miR-N367 shared the same seed sequence to the human hsa-miR-192 targeting the same gene poly(A)-binding protein (PABP) [127]. The examples here reported imply that this viral mechanism represents a more general phenomenon that needs to be fully unraveled.

## 5. Viral Circular RNA

Recently, a novel class of ncRNAs, the Circular RNAs (circRNAs), with gene regulatory functions have been discovered and investigated primarily in gammaherpesviruses [128]. They have initially been considered as incorrect splicing products [129] but recently a master regulatory quality has been suggested for this class of non-coding RNAs [130]. In fact, it has been proposed they act as sponges of miRNAs [19] and thus as regulators of gene expression at post-transcriptional level [131]. In humans, CDR1as is the first example of regulatory circRNA that binds hsa-miR-7 preventing its binding to other molecules [19]. Such inhibitory function is termed “sponge” function.

Several evidences indicated the existence of viral circRNAs encoded in gammaherpesvirus EBV, KSHV and murine gammaherpesvirus 68 (MHV68) [132,133,134,135]. In EBV, more than 30 circRNAs were detected, and these derived from one viral gene that encode for several circRNAs by back-splicing mechanisms [132,134,135]. EBV abundantly expressed circRNAs from the BamHI A rightward transcript (BART) locus (circBARTs) were found mostly expressed during all latency programs (latency type I, type II, and type III) [132] and across tissue and tumor types [134]. Lymphoblastoid cell line obtained with B95-8 EBV strain, a defective EBV virus, thus lacking of miRNA and circBART expression, demonstrates they are not mandatorily required for the maintenance of the EBV genome in cell culture, but since they have been found in different tumor type and PTLD specimens, they could play important role in the viral fitness [132]. It has been shown that one of these circRNAs, EBV cRPMS1, binds human hsa-miR-31, -miR-203, and -miR-451, leading to apoptosis as well as reduced invasiveness. Thus, cRPMS1 sponge activity plays tumorigenic functions in EBV-infected cells [136].

Also in KSHV, at least 10 viral ORFs are reported to express circRNAs [132,133], some of which have oncogenic potential with promotion of cell proliferation [133].

The circRNAs are a very interesting class of ncRNAs thanks to the fine tuning of transcription regulation that might contribute to viral oncogenesis and for this reason they will become surely subject of many studies.

## 6. Conclusions

The scientific interest and attention on v-ncRNAs is constantly expanding. The discovery of new RNA sequences encoded by viruses, due to the newest high-throughput sequencing techniques, is followed by the need of deciphering their functions. Here, we have presented an overview on viruses-encoded ncRNAs and the involved regulatory pathways (Table 2), the mimicry strategies adopted and a novel class of v-ncRNAs, the circRNAs. Interestingly, phylogenetically different viruses seem, on one side to implement common strategies to “tease” the host as “host miRNAs mimicry”, on the other to share common target and pathways, underscoring the importance of key elements in viral persistence, host cell transformation and immune evasion.

## Figures and Tables

**Table 1:** Currently known immune mechanisms and target genes influenced by v-ncRNAs.

Mechanisms Influenced by v-ncRNAs	Target Genes	Molecular Mechanism	v-ncRNAs	References
	PKR, Dicer	PKR and Dicer binding and competitive inhibition	EBERs (EBV) VAI, VA2 (Adenoviruses) TAR-RNA (HIV)	[9]
	IRF4	Downregulation of IRF4-responsive promoter	PAN-RNA (KSHV)	[27]
**IFN Type I Secretion**	STAT2	STAT2 binding and depletion via proteasomal degradation	sfRNA1, sfRNA2 (Zika)	[28,29]
	ISGs	viral RNA-binding ISGs binding and competitive inhibition	sfRNAs (JCV, WNV, Dengue)	[30]
	RIG-1	3′ UTR RIG-I mRNA binding and inhibition of protein expression	ebv-miR-BART6-3p (EBV)	[31]
	CREBBP	3′ UTR CREBBP mRNA binding and degradation induction	ebv-miR-BART16 (EBV)	[33]
	IKKε	3′ UTR IKKε mRNA binding and inhibition of protein expression	kshv-miR-K12-11 (KSHV)	[34]
	IL-6R	mRNA binding and degradation induction	ebv-miR-BART6-3p (EBV) [+ host hsa-miR-197]	[32]
	TWEAKR	3′ UTR TWEAKR mRNA binding and degradation induction	kshv-miR-K12-10 (KSHV)	[35]
**Cytokines Production Impairment**	IL-1R	mRNA binding and and degradation induction	ebv-miR-BHRF-1-2-5p (EBV)	[36]
	IL-1β pathway	3′ UTR NLRP3 mRNA binding	ebv-miR-BART15 (EBV)	[37]
	MyD88	3′ UTR MyD88 mRNA binding and inhibition of protein expression	kshv-miR-K12-5 (KSHV)	[35]
	IRAK1	3′ UTR IRAK1 mRNA binding and inhibition of protein expression	kshv-miR-K12-9 (KSHV)	[35]
	CTSB (MHC-1)	3′ UTR CTSB mRNA binding and degradation induction	ebv-miR-BART2m (EBV)	[38]
	TAP2 (MHC-1)	3′ UTR TAP2 mRNA binding and inhibition of protein expression	ebv-miR-BHRF1-3 (EBV)	[38]
**Antigen Presentation**	LY75 (MHC-1)	3′ UTR LY75 mRNA binding and inhibition of protein expression	ebv-miR-BART1-5p (EBV)	[39]
	IFI30 (MHC-II)	3′ UTR IFI30 mRNA binding and degradation induction	ebv-miR-BART1 (EBV)	[38]
	LGMN (MHC-II)	3′ UTR LGMN mRNA binding and degradation induction	ebv-miR-BART2 (EBV)	[38]
**Neutrophils Chemotaxis**	SP100 (CXCL8)	3′ UTR SP100 mRNA binding and translational repression	MCV-miR-M1-5p (MCPyV)	[40]
	BCL11A, CHD7, ITGAM, RAG-1, TCEA1	In silico analysis	HPV16-miR-H1-1 (HPV)	[41]
	SP3, XRCC4, JAK2, PKNOX1, FOXP1	In silico analysis	HPV16-miR-H2 (HPV)	[41]
	IL12B	3′ UTR IL12B mRNA binding and degradation induction	ebv-miR-BART1, -miR-BART2, -miR-BART10, -miR-BART22, -miR-BHRF1 (EBV)	[38,39]
**T Cells Behaviour**	CXCL11	CXCL11 mRNA binding and inhibition of protein expression	ebv-miR-BHRF1-3 (EBV)	[42]
	Viral T Antigen	ncRNA sequence binding	sv40-miR-S1, jcv-miR-J1 (JCV, BKV, SV40, MCPyV)	[43]
	ASP	Noncoding promoter silencing	HIV-ncRNAs	[45]
	Importin-α5	3′ UTR Importin-α5 mRNA binding and inhibition of protein expression	EBOV-miR-1-5p (Ebola)	[48]
**NK Cells Evasion**	MICB	3′ UTR MICB mRNA binding and inhibition of protein expression	cmv-miR-UL112-1 (CMV), ebv-miR-BART2-5p (EBV), kshv-miR-K12-7 (KSHV)	[49,50,51]
	ULBP3	3′ UTR ULBP3 mRNA binding and protein translation inhibition	Jcv-miR-J1-3p (JCV, BKV)	[52]

**Table 2:** Pathogenetic mechanisms influenced by v-ncRNAs.

Mechanisms Influenced by v-ncRNAs	Target Genes	v-ncRNAs	Reference
	TIA-1	mivaRNAI-138 (Adenovirus)	[73]
	PKR/eIF-2	VAI-RNA (Adenovirus)	[74]
	TGFβ1, SMAD3	LAT (HSV-1)	[75]
	ActD	miR-H3, miR-H4-3p, miR-H4-5p, miR-H24, miR-H19 (HSV-2)	[77]
**Cell Survival (Pro-Apoptotic Factors)**	CASP3, PUMA, p53	EBV-miRNAs	[81,82,83]
	CASP3	kshv-miR-K12-1, -miR-K12-3, -miR-K12-4-3p (KSHV)	[84]
	p21	kshv-miR-K12-1 (KSHV)	[85]
	Mithocondrial Complex -1	β-2.7 (MCPyV)	[86]
	CASP7	sfRNAs (Zika)	[87]
	BclAF1	cmv-miR-UL112-1 (CMV), ebv-miR-BART-17-5p (EBV), kshv-miR-K5 (KSHV)	[88]
	EZH2, DNMT3a, HDAC1	HIV1-ncRNAs	[45,92]
		HSV1-sisRNAs	[93,94]
		CMV-sisRNAs	[95]
		EBV-sisRNA-1	[97]
	JMJD3, UTX, LANA	PAN-RNA (KSHV)	[100,101]
	MAPK	cmv-miR-UL70 (CMV)	[102]
**Virus Infection/Replication**	IE72	cmv-miR-UL112 (CMV)	[103]
	MAPK, TSPYL2, FXYD2, TAOK2, ST7L, TP73	cmv-miR-UL112 (CMV)	[104,105]
	RNF38, NKIRAS2	ebv-miR-BART8-3p, -miR-BART13 (EBV)	[106,107,108]
	MAP3K2	ebv-miR-BART18-5p (EBV)	[109]
	IkB, NFkB	kshv-miR-K1, -miR-K12-1 (KSHV)	[110]
	Rbl2	kshv-miR-K12-4-5p (KSHV)	[111]
		PAN-RNA (KSHV)	[54]
	eIF-2 kinase	EBER1, EBER2 (EBV)	[57,113]
**Cell Transformation**	FOXP1	ebv-miR-BART11 (EBV)	[114]
	PTEN	ebv-miR-BART7-3p (EBV)	[115]
	RNF38	ebv-miR-BART8-3p (EBV)	[107]
	CASTOR1	KSHV-miRNAs	[117]
